# Torsemide Is a More Appropriate Oral Loop Diuretic for Patients with Heart Failure: PRO

**DOI:** 10.34067/KID.0000000000000422

**Published:** 2024-04-09

**Authors:** Conrad J. Macon, David H. Ellison

**Affiliations:** 1 Division of Cardiovascular Medicine, Knight Cardiovascular Institute, Oregon Health and Science University, Portland, Oregon; 2 Division of Nephrology and Hypertension, Department of Medicine, Oregon Health and Science University, Portland, Oregon; 3 Oregon Clinical and Translational Research Institute, Oregon Health and Science University, Portland, Oregon; 4 LeDucq Transatlantic Network of Excellence, Boston, Massachusetts

**Keywords:** chronic heart failure, diuretics

Decongestion with loop diuretics has been foundational to the treatment of both acute and chronic heart failure (CHF) since furosemide's approval in the United States in 1964. During this time, furosemide has been the workhorse oral diuretic being used in 83% of individuals, with torsemide and bumetanide far behind, at 10% and 7%, respectively.^1^ Torsemide is purported to have pharmacokinetic and pharmacodynamic advantages over the other loop diuretics. Whether these perceived advantages translate to benefits in outcome remains unclear. We will argue that the first choice loop diuretic for those with worsening CHF should be torsemide.

The basis of the argument for treating this population with torsemide over furosemide stems from its hypothesized superior pharmacokinetic profile. Torsemide has a high oral bioavailability of approximately 90%–100% (similar to bumetanide) and is not affected by food intake.^2^ Furosemide, on the other hand has an unpredictable and inferior oral bioavailability with a range of approximately 40%–79% (average 60%).^3^ In those with systemic congestion, furosemide's oral bioavailability is even worse.^4^ The duration of action for furosemide is famous as its brand name, Lasix, sounds like its duration of actions (it lasts 6 hours). However, torsemide also has a longer half-life and it is present in the serum for 12 hours which could possibly reduce rebound reabsorption.^3^ In preclinical trials, torsemide inhibited the renin-angiotensin-aldosterone system potentially benefiting those with CHF with less hypokalemia.^2^ The superior oral bioavailability and duration of action give torsemide a hypothetical win in the pharmacokinetic profile in patients with contemporary heart failure.

Until recently, the clinical data also clearly favored torsemide over furosemide. The TOrasemide in Congestive Heart Failure (TORIC) Study was a postmarketing surveillance trial comparing torsemide (*n*=778) and furosemide (*n*=527) in those with New York Heart Association (NYHA) II–III CHF. This open-label, nonrandomized, postmarketing surveillance trial showed a difference in mortality between the individuals treated with torsemide (*n*=17, 2.2%) and those treated with furosemide (*n*=27, 4.5%) (*P* < 0.05). There was also a difference in NYHA classification in those who received torsemide over furosemide (45.8% versus 37.2% *P*-0.00017) with less hypokalemia in the torsemide group as well (12.9% versus 17.9% *P*-0.013).^5^ The limitations to the TORIC trial include its nonrandomized open label design and low use of CHF medications (angiotensin converting enzyme inhibitors 9.5% and *β* blockers 30%).

There are also two randomized controlled trials (RCTs) that favored torsemide over furosemide in those with CHF. The first was an open-label randomized trial evaluating oral torsemide (*n*=113) versus furosemide (*n*=121) in those with heart failure with reduced ejection fraction and on angiotensin converting enzyme inhibitors showed a statistically significant difference in heart failure readmissions (32% versus 17%, *P* < 0.01). The torsemide group also had fewer CHF-related hospital days (106 versus 296 days) and had significant improvement in dyspnea and fatigue scores.^6^ Flaws with this study were its external validity because it was isolated to two centers in Indiana and its open-label design. Importantly, this RCT excluded those with heart failure with preserved ejection fraction.

The other RCT was a multicenter open-label study in Switzerland that randomized those with NYHA II–IV due to CHF to oral torsemide (*n*-122) versus furosemide (*n*=115).^7^ In this study, there was a statistically significant improvement in the NYHA class in the torsemide group but not the furosemide group. The tolerability of the torsemide was also superior to furosemide (global score 2.56 versus 2.22, *P* = 0.0004) with fewer in-hospital days in the torsemide group (95 versus 146 *P* = 0.0006). Neither CHF readmissions (31 versus 35) nor all-cause mortality, however, was altered, perhaps because of low event rate.^7^

The positive results from the nonrandomized open-label TORIC study coupled with the moderately positive results from the smaller RCTs demonstrated the need for a large multicenter randomized trial evaluating the use of furosemide versus torsemide in CHF. The Torsemide Comparison with Furosemide for Management of Heart Failure (TRANSFORM-HF) trial was intended to be this trial. Participants who had an EF of <40% or elevated natriuretic peptide were randomized to torsemide (*n*=1431) versus furosemide (*n*=1428) before discharge with a main outcome measure of all-cause mortality in a time-to-event analysis. Death occurred in 26.1% of those in the torsemide group and 26.2% in the furosemide group (hazard ratio, 1.02 [95% confidence interval (CI), 0.89 to 1.18]). At 12 months, there was also no difference in a composite of all-cause mortality and all-cause hospitalization in either group (47.3 versus 49.3% hazard ratio, 0.92 [95% CI, 0.83 to 102]).^8^ A *post hoc* analysis by TRANSFORM-HF investigators looking at quality-of-life (QOL) metrics, such as Kansas City Cardiomyopathy Questionnaire Clinical Summary Score and Patient Health Questionnaire-2 Score >3, also showed no significant difference between those on discharged on torsemide versus those discharged on furosemide.^9^

Thus, the top line result of the largest clinical trial comparing torsemide and furosemide in heart failure is a negative trial in mortality, rehospitalization, and QOL. Our counterparts will use these results to argue the con case for torsemide in heart failure. Here, we will argue that the trial suffered from major internal validity issues, owing to the design of TRANSFORM-HF Trial as a pragmatic trial.

Pragmatic trials were borne of the criticism that standard RCTs (explanatory trials) are performed at high-volume centers with experienced investigators and select for a small portion of the population limiting their external validity. Explanatory trials also are slow and require significant amount of resources. The goal of pragmatic trials is to demonstrate real-world effectiveness of an intervention in broad patient groups while being quicker and more cost-effective. Transform-HF aimed to achieve these goals by having a broad inclusion criteria (EF <40% or elevated brain natriuretic peptide). They also limited study activities to assignment of the treatment drug. Once participants were discharged from the hospital, management of the study drug was left to the discretion of outpatient clinicians that were not study investigators as per routine care. Follow-up was also pragmatic in that there were no study visits after discharge from the hospital. Data collection was done centrally and remotely *via* telephone calls which were made at 30 days, 6 months, and 1 year and querying the National Death Index at regular intervals.^1^

The design of TRANSFORM-HF as a pragmatic trial did achieve the goals of a pragmatic trial in allowing it to efficiently enroll participants in a cost-effective manner at the peak of the coronavirus disease 2019 pandemic while enrolling a diverse population not often seen in heart failure clinical trials. Yet, although these decisions to pursue a pragmatic design clearly improved the external validity of the trial, they may have affected the trial's internal validity and heavily biased it toward a type 2 error, falsely declaring no difference, when one does indeed exist. As a result of the hands-off approach to the study drug and the remote data collection, the loop diuretic status was only known in 72% (2047/2859) of participants at 1 month. Of those who had a known diuretic status, only 83% (*n*=1710) were on the assigned loop diuretic. In addition, at 1 year there were only visit records for 74.5% (*n*=2131) of individuals, and diuretic status was only known for 64% (*n*=1363) of them. Of those who had diuretic known, only 72.4% (*n*=987) were on the known study drug. Hence, of the 1431 individuals who were assigned torsemide in the hospital, only 510 of them were known to be taking the drug at 12 months. There was also a substantial amount of crossover in the trial, with 7% crossover from torsemide to furosemide at discharge and 3.8% from furosemide to torsemide.

Measurement of secondary study outcomes were also limited by the pragmatic design. Hospitalization events were collected as part of the telephone interviews at 1, 6, and 12 months postdischarge. This information was only collected in 74.5% of individuals at 1 year. Adjudicating this event by relying on a phone interview without hospital records also biases it toward a *β* error. There was a follow-up study looking at QOL markers, such as the Kansas City Cardiomyopathy Questionnaire-CC and the patient health questionnaire-2, which was reported to show no difference in QOL measures.^9^ However, these surveys were only collected in 69.2%, 56.9%, and 46.2% of participants at 1, 6, and 12 months, respectively. The missing data at 1 year clearly increases the risk of selection bias.

Using a 20% reduction in mortality was also overambitious. The evidence that loop diuretics reduce mortality in CHF is very weak with the totality of the evidence being three heterogeneous placebo-controlled trials of 202 participants (3/111 [2.7%] deaths versus 12/110 [10.9%], odds ratio, 0.24; 95% CI, 0.07 to 0.83; *P* = 0.02).^10–13^ The decision to make mortality the main outcome measure stems from a meta-analysis written as a letter to the editor that combined the nonrandomized TORIC study with the before mentioned RCTs (which as previously discussed did not show a mortality benefit).^14^ From the beginning it was unrealistic to expect a 20% reduction in mortality with torsemide over furosemide. It was even less likely to see it in a pragmatic trial in which diuretics were managed by community providers and not by trial investigators resulting in 1/3 of individuals assigned to the intervention arm were known to be taking that intervention at 1 year.

Thus, the noble intentions in choosing to make the largest RCT to date evaluating torsemide a pragmatic trial predisposed it to the null hypothesis from the beginning. The remote follow-up, reliance on community providers, and broad inclusion criteria led to a study in which 34.5% of individuals were known to be taking their assigned study drug and in which secondary outcomes were missing from 25.5% of participants. It is hard to imagine how a drug with perceived benefits as a superior oral diuretic in the outpatient setting would show superiority in a study where it was not used consistently in the outpatient setting.

To summarize, on the one hand, we have a large pragmatic RCT with major internal validity issues that suggested to providers that the use of torsemide did not improve mortality (although it did not hurt it either). On the other hand, there are several smaller explanatory RCTs showing improvement in heart failure hospitalization and dyspnea metrics and a nonrandomized open-label study showing that torsemide may improve survival. Coupled with this, the current published pharmacokinetic data suggest torsemide to have superior gastrointestinal absorption, reliability, and duration of action. The two medications come at a similar cost. With these facts, we will continue to favor torsemide over furosemide in the outpatient setting.

## References

[1] Khan MS Greene SJ Hellkamp AS, . Diuretic changes, Health care resource utilization, and clinical outcomes for heart failure with reduced ejection fraction: from the change the management of patients with heart failure registry. Circ Heart Fail. 2021;14(11):e008351. doi:10.1161/CIRCHEARTFAILURE.121.008351 34674536

[2] Buggey J Mentz RJ Pitt B, . A reappraisal of loop diuretic choice in heart failure patients. Am Heart J. 2015;169(3):323–333. doi:10.1016/j.ahj.2014.12.009 25728721 PMC4346710

[3] Reddy P Mooradian AD. Diuretics: an update on the pharmacology and clinical uses. Am J Ther. 2009;16(1):74–85. doi:10.1097/MJT.0b013e31818d3f67 19142160

[4] Vasko MR Cartwright DB Knochel JP Nixon JV Brater DC. Furosemide absorption altered in decompensated congestive heart failure. Ann Intern Med. 1985;102(3):314–318. doi:10.7326/0003-4819-102-3-314 3970471

[5] Cosin J Diez J.; TORIC investigators. Torasemide in chronic heart failure: results of the TORIC study. Eur J Heart Fail. 2002;4(4):507–513. doi:10.1016/s1388-9842(02)00122-8 12167392

[6] Murray MD Deer MM Ferguson JA, . Open-label randomized trial of torsemide compared with furosemide therapy for patients with heart failure. Am J Med. 2001;111(7):513–520. doi:10.1016/s0002-9343(01)00903-2 11705426

[7] Muller K Gamba G Jaquet F Hess B. Torasemide vs. furosemide in primary care patients with chronic heart failure NYHA II to IV--efficacy and quality of life. Eur J Heart Fail. 2003;5(6):793–801. doi:10.1016/s1388-9842(03)00150-8 14675858

[8] Mentz RJ Anstrom KJ Eisenstein EL, . Effect of torsemide vs furosemide after discharge on all-cause mortality in patients hospitalized with heart failure: the TRANSFORM-HF randomized clinical trial. JAMA. 2023;329(3):214–223. doi:10.1001/jama.2022.23924 36648467 PMC9857435

[9] Greene SJ Velazquez EJ Anstrom KJ, . Effect of torsemide versus furosemide on symptoms and quality of life among patients hospitalized for heart failure: the TRANSFORM-HF randomized clinical trial. Circulation. 2023;148(2):124–134. doi:10.1161/CIRCULATIONAHA.123.064842 37212600 PMC10524905

[10] Burr ML King S Davies HE Pathy MS. The effects of discontinuing long-term diuretic therapy in the elderly. Age Ageing. 1977;6(1):38–45. doi:10.1093/ageing/6.1.38 402800

[11] Myers MG Weingert ME Fisher RH Gryfe CI Shulman HS. Unnecessary diuretic therapy in the elderly. Age Ageing. 1982;11(4):213–221. doi:10.1093/ageing/11.4.213 7180724

[12] Sherman LG Liang CS Baumgardner S Charuzi Y Chardo F Kim CS. Piretanide, a potent diuretic with potassium-sparing properties, for the treatment of congestive heart failure. Clin Pharmacol Ther. 1986;40(5):587–594. doi:10.1038/clpt.1986.228 3533372

[13] Faris RF Flather M Purcell H Poole-Wilson PA Coats AJ. Diuretics for heart failure. Cochrane Database Syst Rev. 2012;(2):CD003838. doi:10.1002/14651858.CD003838.pub3 22336795

[14] Bikdeli B Strait KM Dharmarajan K, . Dominance of furosemide for loop diuretic therapy in heart failure: time to revisit the alternatives? J Am Coll Cardiol. 2013;61(14):1549–1550. doi:10.1016/j.jacc.2012.12.043 23500272 PMC4038646

